# Elevated Serum Immunoglobulin G1 Levels and Left Ventricular Diastolic Dysfunction in Anti‐Centromere Antibody–Positive Patients With Lower Extremity Arterial Disease: A Cross‐Sectional Study

**DOI:** 10.1111/1346-8138.17783

**Published:** 2025-05-16

**Authors:** Tatsuya Shiraki, Hidetaka Kioka, Ikuko Ueda‐Hayakawa, Mitsuyoshi Takahara, Kyoko Tonomura, Aya Maekawa, Yorihisa Kotobuki, Yukihiro Enchi, Minako Ueda, Kosuke Takahari, Yasuharu Takeda, Daisuke Nakamura, Isamu Mizote, Tomohito Ohtani, Manabu Fujimoto, Yasushi Sakata

**Affiliations:** ^1^ Department of Cardiovascular Medicine The University of Osaka Graduate School of Medicine Suita Japan; ^2^ Department of Dermatology The University of Osaka Graduate School of Medicine Suita Japan; ^3^ Department of Laboratory Medicine The University of Osaka Graduate School of Medicine Suita Japan; ^4^ Department of Clinical Laboratory The University of Osaka Hospital Suita Japan; ^5^ Division of Radiology, Department of Medical Technology The University of Osaka Hospital Suita Japan

**Keywords:** anti‐centromere antibody, chronic limb‐threatening ischemia, computed tomography angiography, immunoglobulin G, left ventricular diastolic dysfunction

## Abstract

This cross‐sectional pilot study investigated the clinical characteristics of anti‐centromere antibody (ACA)–positive patients with below‐the‐knee arterial disease. Sixteen ACA‐positive patients (mean age 69 ± 10 years; 94% women) underwent contrast‐enhanced computed tomography evaluation, with arterial damage scored using the Global Limb Anatomic Staging System. Lower extremity arterial disease (LEAD) was defined as a below‐the‐knee arterial score ≥ 1 or > 50% stenosis in above‐the‐knee lesions. Eight patients were categorized into the LEAD group (below‐the‐knee arterial damage score 12 ± 6). The LEAD group showed significantly higher serum IgG1 levels (1029 ± 484 vs. 531 ± 72 mg/dL, *p* < 0.001) and a higher prevalence of diastolic dysfunction (62% vs. 0%, *p* = 0.026) compared to the non‐LEAD group. Patients with diastolic dysfunction had significantly higher serum IgG1 levels than those without (1190 ± 559 vs. 593 ± 139 mg/dL, *p* = 0.008). These findings suggest associations between elevated serum IgG1 levels, below‐the‐knee arterial disease, and left ventricular diastolic dysfunction in ACA‐positive patients.

## 1 Introduction

Anti‐centromere antibodies (ACA) are a specific marker for limited cutaneous systemic sclerosis (lcSSc) [1]. ACA‐positive patients are at an increased risk of developing lower extremity arterial disease (LEAD), even in the absence of skin sclerosis that is typically associated with SSc [2]. Early detection of LEAD in ACA‐positive patients is challenging because the arterial lesions are often limited to the below‐the‐knee regions [3]. Isolated below‐the‐knee arterial disease is characterized by a nonsignificant reduction in the ankle‐brachial index (ABI), which can lead to the development of chronic limb‐threatening ischemia (CLTI) without preceding intermittent claudication [3]. Moreover, ACA‐positive patients with CLTI have a higher risk of major amputation following endovascular therapy for LEAD [4]. This underscores the importance of early assessment of LEAD before the onset of CLTI. However, the prevalence and clinical characteristics of LEAD in ACA‐positive patients remain poorly understood. Accordingly, this study aimed to determine the prevalence of below‐the‐knee arterial disease in ACA‐positive patients using contrast‐enhanced computed tomography (CT) and investigate the clinical characteristics of ACA‐positive patients with LEAD.

## 2 Methods

This cross‐sectional pilot study enrolled 16 consecutive ACA‐positive patients. All patients provided informed consent; the study protocol adhered to the Declaration of Helsinki and was approved by the institutional ethics committee. The diagnosis of SSc was based on the American College of Rheumatology/European League Against Rheumatism (ACR/EULAR) classification criteria [1].

Patients underwent a comprehensive evaluation, including medical examinations, ABI measurements, echocardiography, pulmonary function tests, and contrast‐enhanced CT. Lower extremity arteries were assessed using contrast‐enhanced CT. Above‐the‐knee lesions, from iliac to popliteal arteries, with > 50% stenosis, were defined as significant. Below‐the‐knee arterial damage was scored using an adapted Global Limb Anatomic Staging System for the anterior tibial, peroneal, and posterior tibial arteries (Table S1) [5]. Tibioperoneal trunk damage was considered in both peroneal and posterior tibial artery scores. LEAD was defined as a below‐the‐knee arterial score ≥ 1 or > 50% stenosis in above‐the‐knee lesions. CT evaluations were independently performed by two observers.

Peripheral blood samples were collected for analysis of serum IgG subclasses (BioMajesty 8000 GX; JEOL Ltd., Tokyo, Japan) and T helper 17 cell‐related cytokines (Bio‐PlexPro human Th17 cytokine assays; Bio‐Rad Laboratories Inc., Hercules, CA, USA). The dermatological assessment included the modified Rodnan skin score (mRSS) [6]. Left ventricular diastolic dysfunction was defined according to the American Society of Echocardiography guidelines, requiring ≥ 50% of the following criteria: septal e′ < 7 cm/s, septal E/e′ > 15, left atrium volume index (LAVI) > 34 mL/m^2^, and peak tricuspid regurgitation velocity > 2.8 m/s [7]. Diastolic wall strain (DWS) was calculated as follows: DWS = (PWs – PWd)/PWs, where PWs and PWd indicate the posterior wall thickness at end‐systole and end‐diastole, respectively [8].

Data analysis followed STROBE guidelines. Continuous variables are presented as mean ± standard deviation, categorical variables as *n* (%). Mann–Whitney *U*‐test, Fisher's exact test, and Spearman's correlation coefficient were used, with significance set at *p* < 0.05. Statistical analyses were conducted using R version 4.1.1.

## 3 Results

Contrast‐enhanced CT revealed no cases with > 50% stenosis in above‐the‐knee arteries. Representative three‐dimensional angiographic images and below‐the‐knee arterial damage scores are shown in Figure S1. ACA‐positive patients were categorized into the LEAD (below‐the‐knee arterial damage score ≥ 1, *n* = 8) and non‐LEAD groups (below‐the‐knee arterial damage score = 0, *n* = 8). The ABI in the LEAD group was 1.05 ± 0.09 (right) and 1.07 ± 0.15 (left), compared with 1.12 ± 0.05 (right) and 1.13 ± 0.06 (left) in the non‐LEAD group. In the LEAD group, the mean below‐the‐knee arterial damage score was 12 ± 6, with a higher prevalence of occlusion in the anterior and posterior tibial arteries compared with the peroneal artery (*p* = 0.006, Table S2).

### 3.1 Demographic and Clinical Characteristics

A comparison of demographic characteristics and examination results between the groups is presented in Table 1 and Table S3. Among the 16 patients (94% female), 81% met the ACR/EULAR classification criteria for lcSSc. The remaining 19% did not meet these criteria, but all of them had Raynaud's phenomenon. mRSS‐ and SSc‐specific dermatological findings did not differ between groups. Anti‐Scl‐70 antibodies were positive in 13% (1/8) of both LEAD and non‐LEAD groups, whereas anti‐RNA polymerase III antibodies were negative in all patients in both the LEAD and non‐LEAD groups.

**TABLE 1:** Characteristics of ACA‐positive patients with and without LEAD.

	Overall (*n* = 16)	LEAD group (*n* = 8)	Non‐LEAD group (*n* = 8)	*p*
*Demographic and clinical characteristics*				
Age at the time of CT, years	69 ± 10	76 ± 6	61 ± 9	0.005
Age at the first ACA detection, years	51 ± 13	56 ± 14	46 ± 11	0.11
Duration at the time of CT from the first ACA detection, years	18 ± 12	20 ± 11	16 ± 13	0.53
Diagnosis of limited cutaneous systemic sclerosis	13 (81%)	7 (88%)	6 (75%)	> 0.99
Female sex	15 (94%)	7 (88%)	8 (100%)	> 0.99
Body mass index, kg/m^2^	21 ± 5	21 ± 4	21 ± 6	0.88
Smoking status (past or current)	8 (50%)	4 (50%)	4 (50%)	> 0.99
Hypertension	5 (31%)	5 (62%)	0 (0%)	0.026
Dyslipidemia	5 (31%)	2 (25%)	3 (38%)	> 0.99
Diabetes mellitus	0 (0%)	0 (0%)	0 (0%)	> 0.99
History of malignancy	5 (31%)	4 (50%)	1 (12%)	0.28
Revascularization of coronary artery disease	1 (6%)	0 (0%)	1 (12%)	> 0.99
Revascularization of lower extremity artery disease	2 (13%)	2 (25%)	0 (0%)	0.47
Raynaud's phenomenon	15 (94%)	8 (100%)	7 (88%)	> 0.99
Esophageal involvement	6 (38%)	3 (38%)	3 (38%)	> 0.99
Finger ulcer or gangrene				0.64
No	9 (56%)	4 (50%)	5 (62%)	
Past	2 (13%)	1 (12%)	1 (12%)	
Current	5 (31%)	3 (38%)	2 (25%)	
Digit ulcer or gangrene				0.065
No	10 (63%)	3 (38%)	7 (88%)	
Past	1 (6%)	1 (12%)	0 (0%)	
Current	5 (31%)	4 (50%)	1 (12%)	
Fingertip pitting	5 (31%)	2 (25%)	3 (38%)	> 0.99
Telangiectasias	9 (56%)	5 (62%)	4 (50%)	> 0.99
Nail fold bleeding	6 (38%)	3 (38%)	3 (38%)	> 0.99
mRSS	6 ± 4	5 ± 3	6 ± 5	> 0.99
*Cardiac and respiratory function*				
Echocardiography				
LVDd, mm	41 ± 4	41 ± 4	41 ± 4	> 0.99
LVDs, mm	25 ± 4	25 ± 3	25 ± 5	0.67
LVEF, %	70 ± 8	71 ± 6	69 ± 10	0.56
PWs, mm	14 ± 2	15 ± 2	13 ± 1	0.083
PWd, mm	8.1 ± 2.1	9.2 ± 2.4	7.0 ± 0.8	0.010
DWS, ratio	0.41 ± 0.12	0.37 ± 0.15	0.46 ± 0.04	0.25
E, m/s	0.63 ± 0.16	0.62 ± 0.20	0.64 ± 0.13	0.53
E/A, ratio	0.93 ± 0.39	0.80 ± 0.41	1.07 ± 0.33	0.059
e′, cm/s	6.9 ± 2.2	5.3 ± 1.6	8.2 ± 1.7	0.006
(missing data)	1 (6%)	1 (13%)	0 (0%)	> 0.99
e′ < 7 cm/s	8 (53%)	6 (86%)	2 (25%)	0.041
(missing data)	1 (6%)	1 (13%)	0 (0%)	> 0.99
E/e′, ratio	10 ± 5	13 ± 6	8.1 ± 2.1	0.16
(missing data)	1 (6%)	1 (13%)	0 (0%)	> 0.99
E/e′ > 14	3 (20%)	3 (43%)	0 (0%)	0.077
(missing data)	1 (6%)	1 (13%)	0 (0%)	> 0.99
LAVi, mL/m^2^	33 ± 14	40 ± 5	26 ± 4	0.10
(missing data)	1 (6%)	1 (13%)	0 (0%)	> 0.99
LAVi > 34 mL/m^2^	3 (20%)	3 (43%)	0 (0%)	0.077
(missing data)	1 (6%)	1 (13%)	0 (0%)	> 0.99
TR velocity, m/s	2.2 ± 0.7	2.5 ± 0.4	2.0 ± 0.9	0.27
TR velocity > 2.8 m/s	2 (13%)	2 (25%)	0 (0%)	0.47
(missing data)	1 (6%)	0 (0%)	1 (13%)	> 0.99
Diastolic dysfunction	5 (31%)	5 (62%)	0 (0%)	0.026
Respiratory examination				
%VC, %	100 ± 16	100 ± 19	100 ± 13	0.80
FEV_1.0%_, %	81 ± 10	79 ± 10	82 ± 10	0.51
DLCO, %	70 ± 16	75 ± 20	66 ± 10	0.51
*Serum immunoglobulin and cytokine levels*				
Laboratory data				
Hemoglobin, g/dL	12 ± 1	12 ± 2	12 ± 1	0.87
eGFR, mL/min/1.73m^2^	65 ± 16	59 ± 9	71 ± 20	0.23
NT‐proBNP	238 ± 254	330 ± 317	146 ± 137	0.14
KL‐6, U/mL	239 ± 101	238 ± 57	241 ± 136	0.44
Serum complement, CH50/mL	57 ± 8	60 ± 7	53 ± 7	0.17
Anti‐centromere antibody, U/mL	467 ± 385	497 ± 176	411 ± 331	0.60
Anti‐Scl‐70 antibody	2 (13%)	1 (13%)	1 (14%)	> 0.99
(missing data)	1 (6%)	0	1 (13%)	> 0.99
Anti‐RNA polymerase III antibody	0 (0%)	0 (0%)	0 (0%)	> 0.99
Serum IgG, mg/dL	1377 ± 580	1742 ± 627	1012 ± 155	< 0.001
Serum IgM, mg/dL	108 ± 84	126 ± 113	90 ± 39	0.92
Serum IgA, mg/dL	225 ± 116	290 ± 130	160 ± 46	0.005
T‐cell‐related cytokines				
Interleukin‐1 beta, pg/mL	2.4 ± 0.5	2.3 ± 0.4	2.4 ± 0.6	> 0.99
Interleukin‐17A, pg/mL	4.8 ± 0.7	4.6 ± 0.3	5.0 ± 0.9	0.63
Interleukin‐21, pg/mL	47 ± 4	47 ± 3	46 ± 5	0.67
Interleukin‐23, pg/mL	198 ± 46	194 ± 47	201 ± 48	0.80
Interleukin‐33, pg/mL	15 ± 2	15 ± 2	15 ± 2	0.88
sCD40L, pg/mL	100 ± 103	117 ± 105	84 ± 106	0.44
TNF‐α, pg/mL	4.0 ± 5.6	3.9 ± 3.8	4.0 ± 7.3	0.44

*Note:* Data are presented as number (%) or mean ± standard deviation. The *p* value represents the result of the statistical test comparing each item between the LEAD group and the non‐LEAD group.

Abbreviations: ACA, anti‐centromere antibody; CT, computed tomography; DLCO, diffusing capacity of the lung for carbon monoxide; DWS, distal wall strain; eGFR, estimated glomerular filtration rate; FEV_1.0%_, forced expiratory volume in 1 s; LAVI, left atrial volume index; LEAD, lower extremity arterial disease; LVDd, left ventricular end‐diastolic diameter; LVDs; left ventricular end‐systolic diameter; LVEF, left ventricular ejection fraction; mRSS, modified Rodnan skin score; NT‐proBNP, N‐terminal pro‐brain natriuretic peptide; PWd, posterior wall thickness at end‐diastole; PWs, posterior wall thickness at end‐systole; sCD40L, soluble CD40 ligand; TNF‐α, tumor necrosis factor alpha; TR, tricuspid regurgitation; %VC, % vital capacity.

### 3.2 Cardiac Function

Echocardiography showed preserved systolic function in both groups; however, the prevalence of diastolic dysfunction was significantly higher in the LEAD group (62% vs. 0%, *p* = 0.026). The LEAD group had significantly lower e′ value (*p* = 0.006) and greater left ventricular wall thickness. In the LEAD group, the below‐the‐knee arterial damage score strongly correlated with the e′ (ρ = −0.83, *p* = 0.022) and E/e′ (ρ = 0.83, *p* = 0.021) values, indicating elevated left ventricular filling pressure (Table 2).

**TABLE 2:** Correlation between below‐the‐knee arterial damage score and clinical characteristics.

	ρ	*p*
Age	0.17	0.69
Body mass index	−0.02	0.96
Modified Rodnan skin score	0.19	0.65
LVEF	−0.08	0.84
e′, cm/s	−0.83	0.022
E/e′	0.83	0.021
LAVI	0.56	0.20
TR velocity	−0.61	0.11

Abbreviations: LAVI, left atrial volume index; LVEF, left ventricular ejection fraction; mRSS, modified Rodnan skin score; TR, tricuspid regurgitation.

### 3.3 Serum Immunoglobulin and Cytokine Levels

**FIGURE 1:**
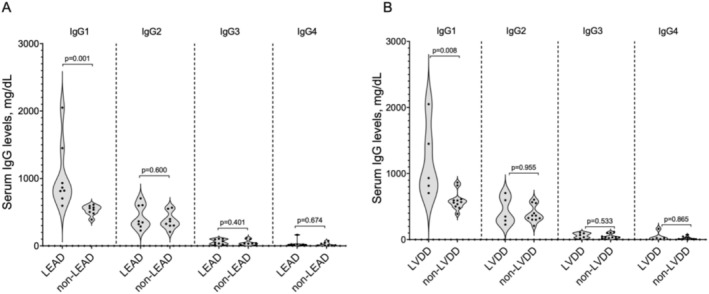
Serum levels of IgG subclasses. (A) The differences in serum levels of IgG subclasses between the LEAD group (*n* = 8) and non‐LEAD group (*n* = 8). (B) The differences in serum levels of IgG subclasses between patients with left ventricular diastolic dysfunction (*n* = 5) and those without (*n* = 11). The dark bold dashed line represents the median, the dark light dashed lines indicate the 25% and 75% quartiles, and the black dots represent individual data. LVDD, left ventricular diastolic dysfunction.

The LEAD group showed significantly higher serum IgG (*p* < 0.001) and IgA levels (*p* = 0.005). IgM levels showed no significant difference between the groups (*p* = 0.92). Among IgG subclasses, only IgG1 levels were significantly higher in the LEAD group (1029 ± 484 mg/dL vs. 531 ± 72 mg/dL, *p* = 0.001, Figure 1A). Patients with left ventricular diastolic dysfunction had significantly higher serum IgG1 levels than those without (1190 ± 559 mg/dL vs. 593 ± 139 mg/dL, *p* = 0.008, Figure 1B). There was no significant difference in serum ACA levels between the LEAD group (497 ± 176 U/mL) and the non‐LEAD group (411 ± 331 U/mL) (*p* = 0.60), and no correlation was observed between IgG1 levels and serum ACA levels (ρ = −0.20, *p* = 0.47). No significant differences were observed in T‐cell‐related cytokines between groups (Table 1). Other cytokines (interferon‐γ, interleukin‐4, interleukin‐6, interleukin‐10, interleukin‐17F, interleukin‐22, interleukin‐25, and interleukin‐31) were below the detection threshold of the assay.

## 4 Discussion

In this cross‐sectional pilot study, half of the consecutive ACA‐positive patients had LEAD, predominantly in below‐the‐knee arteries. ACA‐positive patients with LEAD had concurrent left ventricular diastolic dysfunction. Elevated serum IgG1 levels were significantly higher in patients with both conditions.

Despite normal ABI, contrast‐enhanced CT revealed below‐the‐knee arterial disease in ACA‐positive patients, particularly occlusion in anterior and posterior tibial arteries, consistent with previous studies [4]. The below‐the‐knee arterial damage score correlated with the E/e′ and e′ values in echocardiographic findings. Left ventricular diastolic dysfunction was observed exclusively in the LEAD group, which is clinically significant as it predicts mortality in systemic sclerosis patients [9]. Coronary microvascular dysfunction, a known contributor to left ventricular diastolic dysfunction [10], has been demonstrated in SSc patients using stress cardiac magnetic resonance imaging and invasive intracoronary pressure wire studies [11]. The association between LEAD and diastolic dysfunction may be explained by endothelial dysfunction, known features of systemic sclerosis [12].

Patients with LEAD showed significantly elevated serum IgG and IgA levels, aligning with a previous study that reported an association between these immunoglobulins and cardiovascular events [13]. Notably, only serum IgG1 levels were significantly higher in patients with both LEAD and left ventricular diastolic dysfunction. Previous research has not explored the relationship between IgG subclasses and vascular complications in systemic sclerosis [14]. Our findings suggest that serum IgG1 levels may increase specifically in the presence of LEAD in ACA‐positive patients, providing new insight into the potential role of IgG1 in SSc‐related vascular complications, as well as in left ventricular diastolic dysfunction. Furthermore, elevated IgG1 levels have been reported in patients with left ventricular diastolic dysfunction [15]. These observations indicate a possible pathway where increased IgG1 contributes to endothelial dysfunction, leading to both LEAD and diastolic dysfunction in ACA‐positive patients.

The primary limitation of this study is its small sample size, which restricts our ability to draw definitive conclusions and perform robust multivariate analyses. The cross‐sectional design limits causal inference between elevated IgG1 levels and the development of LEAD and left ventricular dysfunction. The lack of a control group limits our ability to determine the specificity of our findings to ACA‐positive patients. Future studies should address these limitations by enrolling more participants, incorporating a longitudinal design, directly assessing endothelial function, including appropriate control groups, and performing comprehensive multivariate statistical analyses.

In conclusion, this cross‐sectional pilot study revealed that half of consecutive ACA‐positive patients developed LEAD, with left ventricular diastolic dysfunction significantly prevalent among those in the LEAD group. Our findings suggest a possible association between elevated serum IgG1 levels and both below‐the‐knee arterial disease and left ventricular diastolic dysfunction in ACA‐positive patients.

## Ethics Statement

The study protocol adhered to the Declaration of Helsinki and was approved by the institutional ethics committee (No. 21114).

## Consent

All patients provided informed consent.

## Conflicts of Interest

The authors declare no conflicts of interest.

## Supporting information


Data S1.
