# Supplementary material for: Elevated Serum Immunoglobulin G1 Levels and Left Ventricular Diastolic Dysfunction in Anti‐Centromere Antibody–Positive Patients With Lower Extremity Arterial Disease: A Cross‐Sectional Study
Source: J Dermatol. 2025 May 16;52(6):1070–7. doi: 10.1111/1346-8138.17783 (PMC12149368; doi:10.1111/1346-8138.17783)
Supplement: Supplementary file 1 — Data S1. [file JDE-52-1070-s001.docx]

Supplemental Figure 1. Representative cases for below-the-knee arterial damage score.


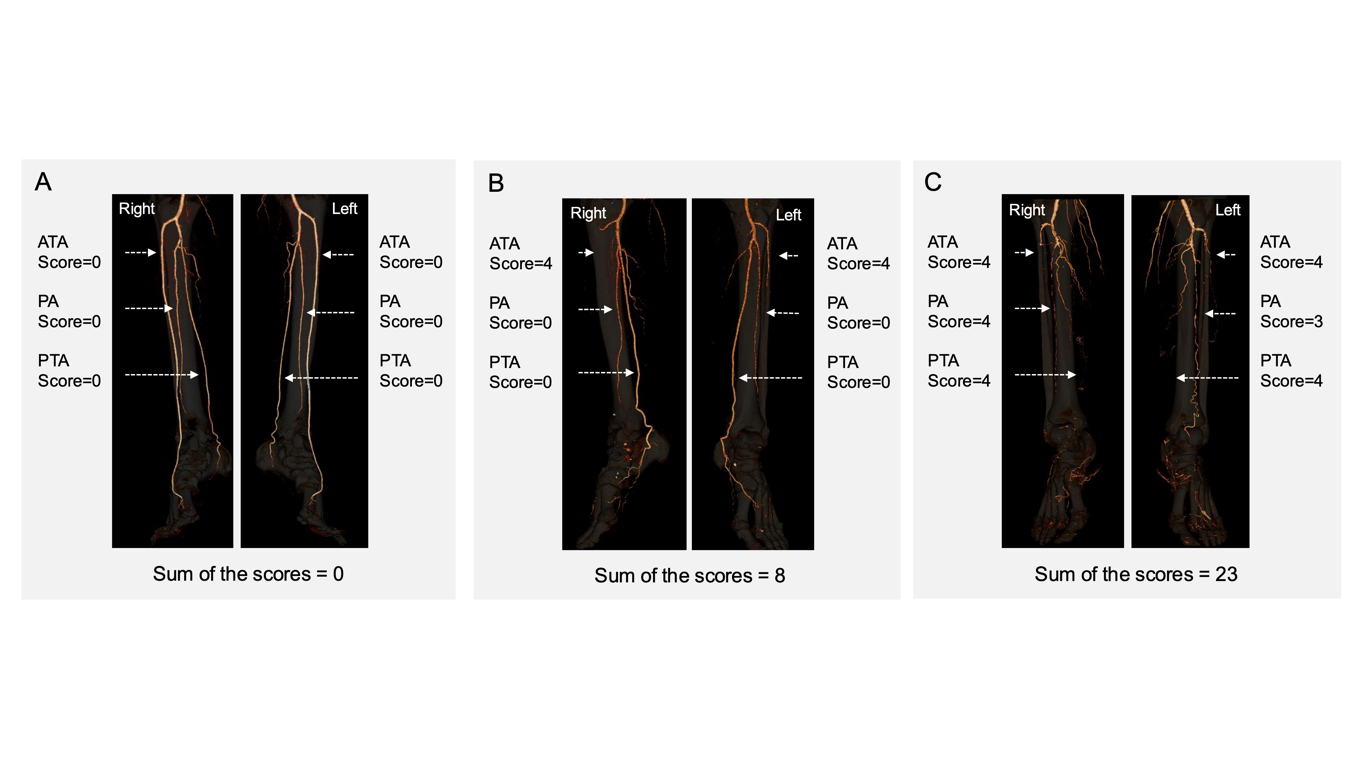


Representative contrast-enhanced computed tomography images and below-the-knee arterial damage scores are shown. The below-the-knee arterial damage scores are summarized, with the score evaluated for anterior tibial, peroneal, and posterior tibial arteries. The assigned below-the-knee arterial damage scores are as follows: 0 for case (A), 8 for case (B), and 23 for case (C). ATA, anterior tibial artery; PA, peroneal artery; PTA, posterior tibial artery.

Supplemental Table 1. The definition of below-the-knee arterial damage score

| Below-the-knee arterial damage score | |
| --- | --- |
| 0 | Mild or no significant (<50%) disease |
| 1 | Focal stenosis less than 3 cm |
| 2 | Total length of artery disease less than 10 cm  Single focal occlusion less than 3 cm |
| 3 | Total length of artery disease between 10 to 20 cm  Total length of occlusion between 3 to10 cm |
| 4 | Total length of artery disease more than 20 cm  Total occlusion length more than 10 cm |

Supplemental Table 2. Below-the-knee arterial disease in ACA-positive patients with LEAD

|  | ATA | PA | PTA | P value |
| --- | --- | --- | --- | --- |
| Arterial damage score |  |  |  | 0.11 |
| 0 | 20 (63%) | 27 (84%) | 19 (63%) * |  |
| 1 | 0 (0%) | 0 (0%) | 0 (0%) * |  |
| 2 | 1 (3%) | 3 (9%) | 1 (3%) * |  |
| 3 | 2 (6%) | 1 (3%) | 1 (3%) * |  |
| 4 | 9 (28%) | 1 (3%) | 9 (30%) * |  |
| Hypoplasia | 0 (0%) | 0 (0%) | 2 (6%) | 0.13 |
| Occlusion | 9 (28%) | 0 (0%) | 7 (23%) * | 0.006 |
| Calcification | 3 (3%) | 5 (5%) | 0 (0%) * | 0.08 |

Data are presented as number (%). * In calculating the percentage, hypoplastic arteries in the PTA (n=2) were excluded. ACA, anti-centromere antibody; ATA, anterior tibial artery; LEAD, lower-extremity arterial disease; PA, peroneal artery; PTA, posterior tibial artery.

Supplemental Table 3. Medication usage in ACA-positive patients with and without LEAD

|  | Overall  (n = 16) | LEAD group (n = 8) | Non-LEAD group (n = 8) | p value |
| --- | --- | --- | --- | --- |
| Medication |  |  |  |  |
| Aspirin | 3 (19%) | 3 (38%) | 0 (0%) | 0.054 |
| P2Y12 inhibitor | 2 (13%) | 1 (13%) | 1 (13%) | > 0.99 |
| Sarpogrelate Hydrochloride | 9 (56%) | 5 (63%) | 4 (40%) | 0.61 |
| ACE inhibitor or ARB | 2 (13%) | 2 (25%) | 0 (0%) | 0.13 |
| Calcium channel blocker | 5 (31%) | 4 (50%) | 1 (13%) | 0.11 |
| Nitrates | 3 (19%) | 2 (25%) | 1 (13%) | 0.52 |
| Statin | 3 (19%) | 1 (13%) | 2 (25%) | 0.52 |
| Prednisolone | 2 (13%) | 0 (0%) | 2 (25%) | 0.13 |
| Prostacyclin | 7 (44%) | 3 (38%) | 4 (50%) | 0.61 |
| Bosentan | 5 (31%) | 3 (38%) | 2 (25%) | 0.59 |

Data are presented as number (%). ACE, Angiotensin-Converting Enzyme; ARB, Angiotensin II Receptor Blocker.
